# Multi-omics insights implicate the remodeling of the intestinal structure and microbiome in aging

**DOI:** 10.3389/fgene.2024.1450064

**Published:** 2024-11-12

**Authors:** Shaohua Chen, Chengbang Wang, Xiong Zou, Hanwen Li, Guanglin Yang, Xiaotao Su, Zengnan Mo

**Affiliations:** ^1^ Department of Urology, Guangxi Medical University Cancer Hospital, Nanning, Guangxi, China; ^2^ Center for Genomic and Personalized Medicine, Guangxi key Laboratory for Genomic and Personalized Medicine, Guangxi Collaborative Innovation Center for Genomic and Personalized Medicine, Guangxi Medical University, Nanning, Guangxi, China; ^3^ Department of Neurology, First Affiliated Hospital of Guangxi Medical University, Guangxi Medical University, Nanning, Guangxi, China; ^4^ Institute of Urology and Nephrology, First Affiliated Hospital of Guangxi Medical University, Guangxi Medical University, Nanning, Guangxi, China

**Keywords:** aging, inflammaging, gut microbiota, metabolism, intestinal epithelial cells

## Abstract

**Background:**

Aging can impair the ability of elderly individuals to fight infections and trigger persistent systemic inflammation, a condition known as inflammaging. However, the mechanisms underlying the development of inflammaging remain unknown.

**Methods:**

We conducted 16S rRNA sequencing of intestinal contents from young and old C57BL/6J mice to elucidate changes in gut microbiota diversity and microbial community composition after aging. Aging-related differential bacterial taxa were then identified, and their abundance trends were validated in human samples. The variances in intestinal barrier function and circulating endotoxin between groups were also assessed. Furthermore, widely targeted metabolomics was conducted to characterize metabolic profiles after aging and to investigate the key metabolic pathways enriched by the differential metabolites.

**Results:**

Our findings demonstrated an increase in relative proportion of pathogenic bacteria with age, a trend also revealed in healthy populations of different age groups. Additionally, aging individuals exhibited reduced intestinal barrier function and increased circulating endotoxin levels. Widely targeted metabolomics revealed a significant increase in various secondary bile acid metabolites after aging, positively correlated with the relative abundance of several aging-related bacterial taxa. Furthermore, old group had lower levels of various anti-inflammatory or beneficial metabolites. Enrichment analysis identified the starch and sucrose metabolism pathway as potentially the most significantly impacted signaling pathway during aging.

**Conclusion:**

This study aimed to provide insights into the complex interactions involved in organismal inflammaging through microbial multi-omics. These findings lay a solid foundation for future research aimed at identifying novel biomarkers for the clinical diagnosis of aging-related diseases or potential therapeutic targets.

## Introduction

The incidence of various tumors and chronic diseases grows along with the aging process and longer life expectancy. Previous research has shown that aging individuals have a systemic, chronic, non-infectious inflammatory state characterized by elevated levels of pro-inflammatory cytokines in the systemic and tissue compartments, as well as sustained chronic immune system activation (Ferrucci and Fabbri, 2018), known as inflammaging, which is linked with onset of various aging-related phenotypes and degenerative diseases (Franceschi et al., 2018). Inflammaging arises from multifactorial processes involving several physiological systems, including the immune, endocrine, digestive, and urinary systems (Dugan et al., 2023). While extensive research has focused on the molecular mechanisms of inflammaging, the traditional experimental approach has impeded comprehensive and objective characterization of the complex interactions between different organs. Furthermore, the communication mechanisms and regulatory networks among different organs have been frequently disregarded, resulting in an inaccurate portrayal of the overall landscape of inflammaging, contributing to the limited applicability of these findings in clinical settings.

The human gut microbiota constitutes a vast and highly specific habitat composed of trillions of bacteria (Santoro et al., 2018). The gut microbiota and host have a stable mutualistic interaction. The host provides a safe habitat for gut microbiota, while gut microbiota facilitates nutrient absorption required by the host through fermentation and prevents pathogen colonization (Ragonnaud and Biragyn, 2021). However, compelling evidence has revealed the alterations in the gut microbiota in aging individuals, with a drop in the abundance of beneficial bacteria or an increase in proportion of specific microbial taxa with pro-inflammatory phenotypes (Bodogai et al., 2018). The human intestine harbors approximately 3.9 × 10^13^ bacteria, which is equivalent to the total number of human cells, and the gut microbiota has tens of millions of genes, a hundred times more than the genes in the human genome (Qin et al., 2010). The majority of these genes are non-redundant microbial genes enriched in various primary metabolic pathways, demonstrating the high metabolic potential of the gut microbiota (Tierney et al., 2019). The metabolites produced by the gut microbiota can directly influence the local microenvironment and modulate organ functions, taking a crucial stage in maintaining internal homeostasis as well as immune regulation (Wilmanski et al., 2021), as well as forging a reciprocal connection with intestinal epithelial cells (Funk et al., 2020). Often, aging is accompanied by metabolome changes, which is closely linked to neurocognitive dysfunction and various aging-related diseases (Yoshimoto et al., 2021). However, the systemic alterations in inflammaging and their correlation with the gut microbiota remains to be elucidated. Indeed, research in this field has the potential to advance precision medicine by deepening the understanding of gut microbiomics, discovering novel biomarkers for clinical diagnosis, and identifying potential therapeutic targets.

Herein, we conducted a systematic analysis of gut microbiota and their functions across different age groups by 16S rRNA sequencing, delving into the mechanism involving inflammaging from standpoint of gut microbiota, and shedding light on age-related changes in gut structure. Furthermore, using widely targeted metabolomics analysis, we investigated variations in the metabolic profile of gut microbiota between groups, providing an overview of the differential metabolites detected after aging. In a nutshell, we elucidated the mechanism of inflammaging using the gut microbiota as a focal point, laying a solid foundation for the discovery of novel biomarkers for age-related diseases and the exploration of potential therapeutic targets.

## Materials and methods

### Collection and processing of samples

**FIGURE 1:**
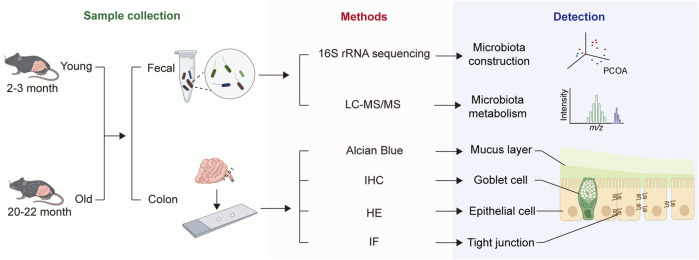
Overall workflow of the study.

After 1 week of adaptation feeding, 15 mice, consisting of 8 young and 7 old, were euthanized for blood sampling from their orbits, which were stored at room temperature for 30 min, and then centrifuged at 3,000 rpm for 20 min at 4°C to extract plasma, which was stored at −80°C for subsequent experiments. The abdominal skin of the mice was then incised to fully expose and open the lower digestive tract, from cecum to anus, and the length from colon to anus was measured. The intestinal segment was excised, placed in Carnoy’s fixative solution for alcian blue staining. Next, feces from the colon were collected for subsequent sequencing. Finally, the intestinal segments were longitudinally cut and immersed in PBS to thoroughly wash away feces before being embedded in formalin fixative for hematoxylin-eosin (HE), immunofluorescence (IF), and immunohistochemistry (IHC) staining. The study workflow is depicted in Figure 1.

### Paraffin embedding and HE staining

Fresh colon tissues were preserved in paraformaldehyde fixative and then transferred to a dehydration chamber. The tissues were dehydrated using a series of alcohol gradients, followed by two 10-minute washes in xylene. Subsequently, the tissues were immersed in molten paraffin, cooled, solidified, and sliced into four-μm thick sections using a microtome. The sections were floated on warm water to flatten, transferred to glass slides, and oven-dried. The sections were then immersed in two different xylene solutions for 20 min each, followed by incubations in 100% ethanol and 75% ethanol, respectively. The sections were stained in hematoxylin solution, differentiated in 1% hydrochloric acid ethanol, counterstained with eosin for 5 min, and dehydrated in alcohol.

### Alcian blue staining

To preserve the integrity of the mucus layer, only colon segments between consecutive feces were collected and fixed in Carnoy’s fixative for 48 h. Next, the samples were immersed in two different methanol solutions for one hour each, followed by two separate one-hour immersions in 100% ethanol. The samples were incubated in xylene and embedded in paraffin. Paraffin blocks were and dried in the oven. The sections were then sequentially immersed in two different xylene solutions for 20 min each, followed by 5-min incubations in 100% ethanol and 75% ethanol, respectively, then rinsed with water. The sections were stained in alcian blue solution A for 15 min and alcian blue solution B for 3 min, rinsed with water, dehydrated, and mounted with neutral resin for microscopy examination.

### IHC and IF staining

Tissue sections were deparaffinized in xylene and ethanol for IHC staining. Antigen retrieval was performed in boiling EDTA buffer, and the sections were blocked. The sections were incubated with primary antibodies, followed by secondary antibodies labeled with biotin and peroxidase-labeled streptavidin. Tissue slices were treated identically for IF staining, then incubated with fluorescent-labeled secondary antibodies and counterstained with DAPI. As a final step, the sections were mounted on anti-fluorescence mounting medium.

### Enzyme-linked immunosorbent assay (ELISA)

As described previously, lipopolysaccharide (LPS) and lipopolysaccharide-binding protein (LBP) concentrations in plasma were quantified using ELISA (Su et al., 2024). Standards were prepared, and samples were diluted correspondingly before loading onto an ELISA plate. Color development was induced following incubation and washing steps.

### 16S rRNA sequencing data processing and analysis

16S rRNA sequencing data were first extracted from raw reads. The barcodes and primer sequences were removed, and reads from each sample were concatenated to generate raw tags. Subsequently, low-quality and short-length sequences were filtered out to generate clean tags (CTs). These CTs were matched with species annotation databases to remove chimeric sequences, yielding final effective tags (ETs). Next, the UPARSE algorithm was employed to cluster ETs from all samples into operational taxonomic units (OTUs) with 97% sequence similarity. Taxonomic annotation at different levels was performed using Mothur method and SILVA138 database. The data was normalized, and the relative abundances of microbial communities at different taxonomic levels were visualized using the “ggplot2”, “ggalluvial”, and “reshape2” R packages. Following that, alpha and beta diversity between different samples was calculated using the QIIME software.

Differential microbial community identifications at various taxonomic levels were analyzed and visualized using Welch’s *t*-test and the Linear Discriminant Analysis Effect Size (LEfSe) analysis. The differential microbial taxa were validated using human gut microbiota data from the GMrepo database, which comprises 71,642 human gut microbiome data from 353 datasets, including 132 human-related phenotype data (Wu et al., 2020). The PRJEB11419 dataset was used for validation, which is part of the American Gut Project (AGP) and includes data from 11,336 volunteers across 42 countries or regions, including the United States, the United Kingdom, and Australia (McDonald et al., 2018). To minimize effect of health conditions, past diseases, and medication history on microbial abundance, we further screened the samples in the dataset using the following criteria: (a) healthy volunteers without inflammatory bowel disease or diabetes; (b) no antibiotic usage over the past year; (c) availability of complete personal information data; (d) 16S rRNA sequencing data meeting quality control standards.

Besides, to perform a more rigorous validation of differential abundance results at the genus level, the “LinDA” (Linear Models for Differential Abundance Analysis) R package was employed as a supplementary tool to support findings from Welch’s *t*-test and LEfSe, specifically addressing concerns regarding high false-positive rates associated with methods lacking integrated multiple testing correction. LinDA was configured with a significance threshold set at α = 0.05, a prevalence cutoff at 0.1, and a library size cutoff of 1,000 to exclude low-abundance OTUs. Additionally, Winsorization was applied to minimize the influence of outliers on the dataset, enhancing result reliability. This approach allows for a comprehensive assessment of genera by leveraging LinDA’s multiple testing correction capabilities, thus complementing the findings obtained through traditional methods.

### Sample handling and extraction for widely targeted metabolomics analysis

Samples of intestinal contents (20 mg) were transferred to pre-labeled centrifuge tubes after multi-point sampling. The samples were homogenized with a steel ball at 30 Hz for 20 s and centrifuged at 3,000 rpm for 30 s at 4°C. Next, 400 μL of 70% methanol-water extraction solution containing internal standards was added to ensure consistent extraction efficiency across samples. After vortexing at 1,500 rpm for 5 min, the samples were centrifuged at 12,000 rpm and 4°C for 10 min. The resulting supernatant was transferred to new centrifuge tubes and stored at −20°C for 30 min. Finally, the supernatant was centrifuged again at 12,000 rpm for 3 min at 4°C before analysis.

### Chromatography and mass spectrometry acquisition parameters

The instrumentation system used in this study consisted of ultra-performance liquid chromatography (UPLC) coupled with tandem mass spectrometry (MS/MS). Qualitative analysis was conducted using a self-built targeted standard database that included over 3,000 metabolites from various chemical groups, including amino acids, organic acids, nucleotides, carbohydrates, lipids, phenolic compounds, vitamins, and bile acids. These metabolites were used to establish theoretical Q1 (MS1), Q3 (MS2), and retention time (RT) databases. Subsequently, biological samples were directly analyzed qualitatively and quantitatively based on the information stored in the databases. Metabolite identification was based on RT, and precursor-product ion pair information. Quantification was performed using the multiple reaction monitoring (MRM) mode of triple quadrupole mass spectrometry, which allowed for precise detection and quantification of target metabolites by filtering out non-target ions. Chromatographic peak integration and calibration were performed using MultiQuant software, with each peak area reflecting the relative content of the respective substance.

### Data processing and analysis of metabolomics

Metabolomics data was preprocessed and analyzed using the “MetaboAnalystR” package. Firstly, the relative metabolite content data matrix was standardized using the Normalization function. Following that, principal component analysis (PCA) was conducted to examine the sample distribution. Orthogonal partial least squares discriminant analysis (OPLS-DA) was used to determine the significance of each metabolite’s variable significance in the projection (VIP) value and determine overall differences across groups. Subsequently, differential metabolites were determined using VIP ≥1 and absolute Log_2_fold change (Log_2_FC) ≥ 1.

Furthermore, in our quest to explore the correlation between gut microbiota and metabolites, we embarked on a correlation analysis. Initially, we pinpointed metabolites that exhibited significant differences and extracted the relevant data for the analysis. We then harnessed the rcorr function from the “Hmisc” package to compute the correlation matrix and its *P* values, applying the Bonferroni method for multiple testing correction. Finally, we visualized the significant correlations using “ggplot2” to generate a heatmap that displays the relationships between the differential microbiota and metabolites. Here, the differential microbiota refers to the two genera identified by LEfSe and the intersection between those identified by LinDA and Welch’s *t*-test.

### Statistical analysis

Student’s t-test or Welch’s *t*-test was used for normally distributed data, whereas Wilcoxon rank-sum test was used for non-normally distributed data. *P* < 0.05 was considered for statistically significant.

## Results

### Alpha and beta diversity of gut microbiota diversity in young and old mice

**FIGURE 2:**
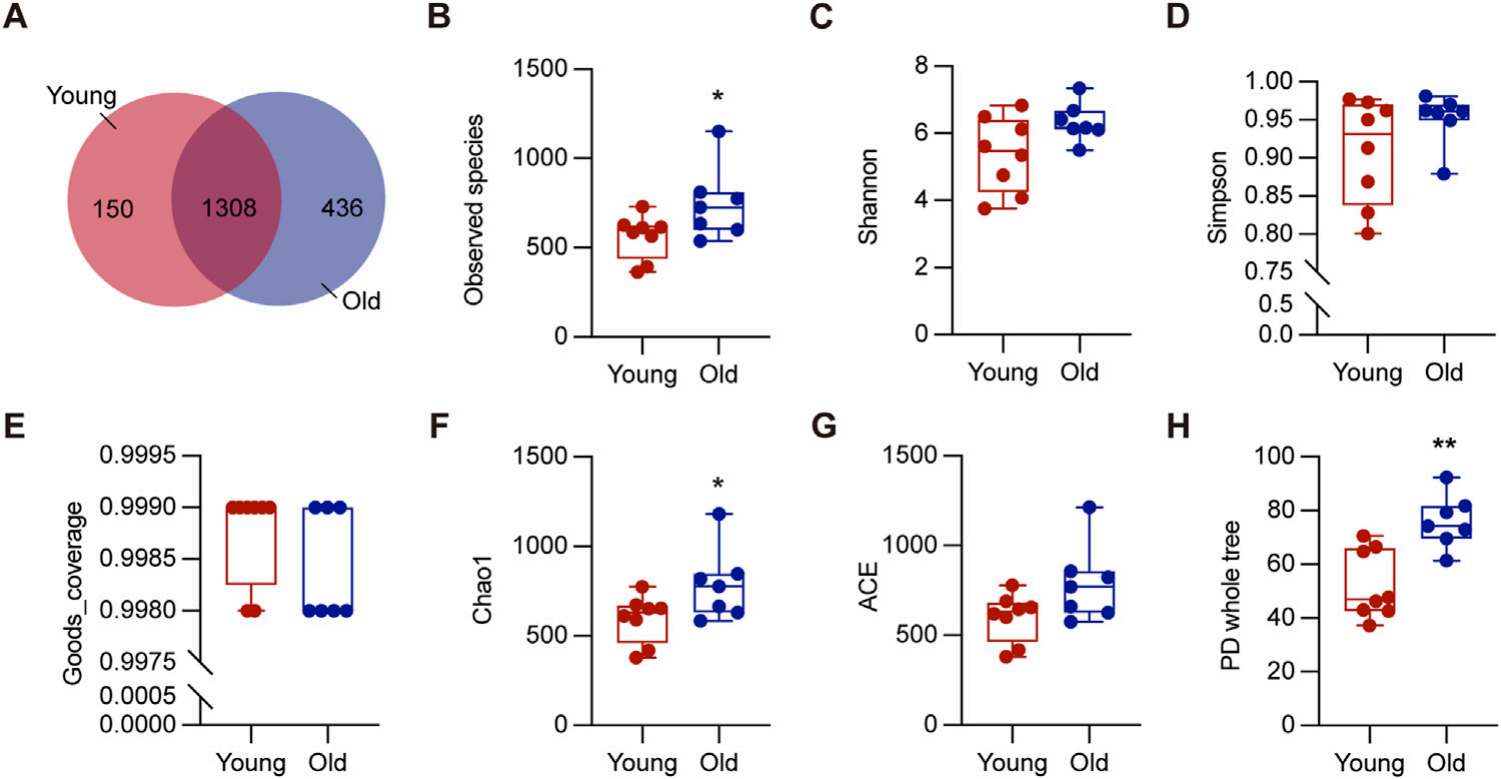
Alpha diversity of gut microbiota in young and old groups. **(A)** The Venn diagram displayed overlapping and distinct OTUs between groups. **(B–H)** Comparisons of alpha diversity of gut microbiota in the intestinal contents between groups. *P < 0.05, and **P < 0.01.

To investigate the effect of aging on gut microbiota, we collected colon contents from a total of 15 mice, consisting of 8 young (2–3 months) and 7 old (20–22 months), for 16S rRNA sequencing. These ages correspond to approximately 20 and 60-year-old of chronological age in humans, respectively (Dutta and Sengupta, 2016). As depicted in Figure 2A, 1744 OTUs were detected in the old mice, while 1,458 OTUs in the young mice, and 1,308 OTUs were shared by both groups. The rarefaction curves plateaued when the number of species retrieved from each sample was maximum, suggesting adequate sequencing depth (Supplementary Figure S1A). We analyzed richness and diversity of gut microbiota communities in different ages using α-diversity, including observed species, Chao1, Simpson, PD whole tree, ACE, goods coverage, and Shannon (Figures 2B–H). The old group outperformed the young group in terms of Observed species, Chao1, and PD whole tree indices (*P* < 0.05), suggesting increased microbial diversity and community richness (Fu et al., 2020). These results demonstrated that aging significantly increased richness and diversity within the microbiota, consistent with previous research (Hoffman et al., 2017).

**FIGURE 3:**
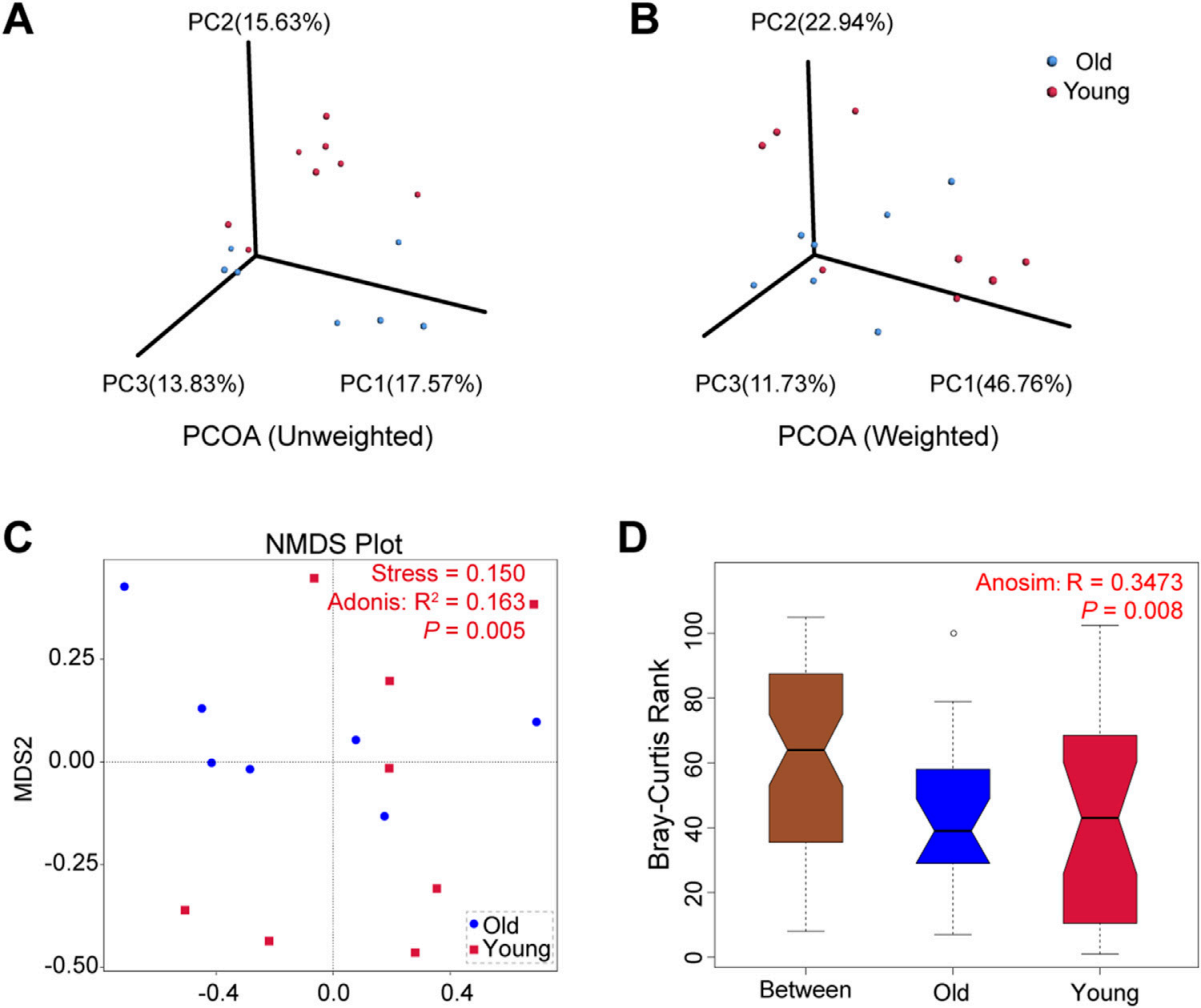
Beta diversity of gut microbiota in young and old groups. **(A)** PCoA based on unweighted UniFrac distances. **(B)** PCoA based on weighted UniFrac distances. **(C)** NMDS analyses based on Bray-Curtis distance values (Adonis: *R* ^2^ = 0.163, *P* = 0.005). **(D)** Anosim based on Bray-Curtis distance (R = 0.3473, *P* = 0.008).

The microbial community composition between groups was compared using beta diversity. We conducted principal co-ordinates analysis (PCoA) based on the UniFrac distances and selected the first three principal coordinate combinations for visualization. Figures 3A, B depict PCoA visualizations based on unweighted and weighted UniFrac distances, respectively, demonstrating differences between samples from young and old groups. Similarly, non-metric multi-dimensional scaling (NMDS) analysis revealed that old mice exhibited a more concentrated distribution, while samples from the young group showed relatively dispersed clusters with a stress of 0.150 (Figure 3C). The subsequent Adonis analysis demonstrated significant differences in microbial composition between groups (*R* ^2^ = 0.163, *P* = 0.005). Furthermore, Anosim analysis using Bray-Curtis distance values compared microbial community structures in samples, yielding statistically significant differences between groups (R = 0.3473, *P* = 0.008) (Figure 3D). These findings suggested that aging altered gut microbiota composition in mice, with high individual variation between samples in the young mice.

### Microbial composition at phylum and genus levels in young and old mice

**FIGURE 4:**
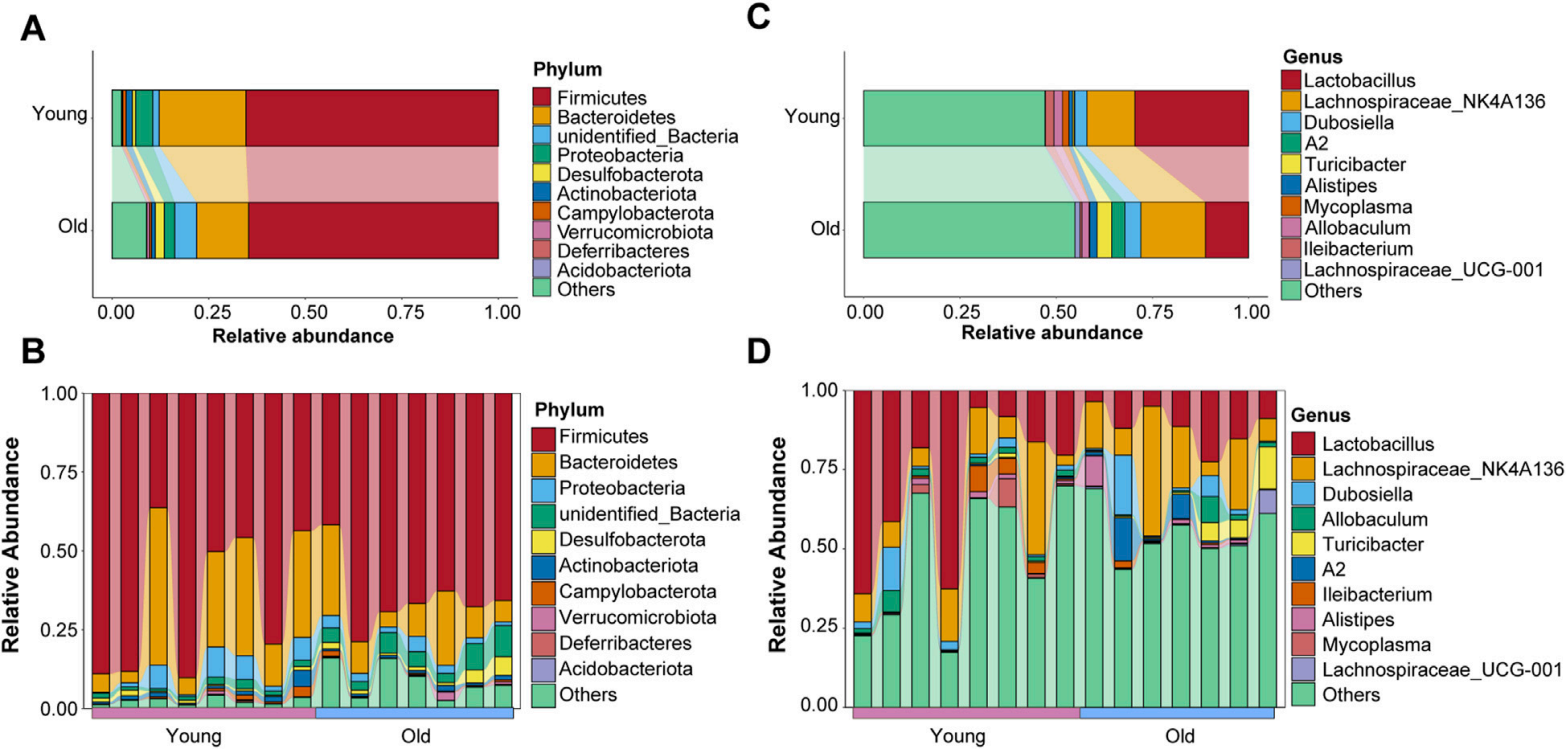
Microbial composition of gut microbiota at the phyla level of old and young groups. **(A)** The top ten phyla abundance in the old and young groups. **(B)** The top ten phyla abundance in each sample. **(C)** The top ten genera abundance in the old and young groups. **(D)** The top ten genera abundance in each sample.

Taxonomic information on microbiota was retrieved using representative OTU sequences. Firmicutes, Bacteroidetes, Proteobacteria, Desulfobacterota, and Actinobacteriota were the top five abundant phyla, with the relative abundance of top ten taxa in both groups visualized using a Sankey diagram (Figure 4A). Interestingly, compared to the young group, relative abundance of Bacteroidetes decreased while that of Desulfobacterota increased in the old group, which is consistent with previous research on age-related changes in gut microbial profiles in crab-eating macaques (Wei Z. Y et al., 2022). The relative abundance ranking of the top 10 taxa at the phylum level in each sample from the young and old groups was also analyzed as depicted in Figure 4B, with Firmicutes and Bacteroidetes being the predominant phyla. Koren and colleagues found significant transitions in the ratio of Firmicutes to Bacteroidetes with aging (Binyamin et al., 2020). However, our results revealed otherwise (Supplementary Figure S1B).

In addition, we evaluated the distribution characteristics of gut microbiota at the genus level. *Lactobacillus*, *Lachnospiraceae_NK4A136*, *Dubosiella*, *Allobaculum*, *Turicibacter*, *A2*, *Ileibacterium*, *Alistipes*, *Mycoplasma*, and *Lachnospiraceae_UCG-001* were among the genera with relatively high relative abundance. *Lactobacillus* and *Lachnospiraceae_NK4A136* were the most abundant genera in the gut microbiota of young and old mice, respectively (Figure 4C). The relative abundance ranking of the top 10 taxa at the genus level in each sample from the young and old groups is depicted in Figure 4D.

### Identification and validations of differential microbiota taxa

**FIGURE 5:**
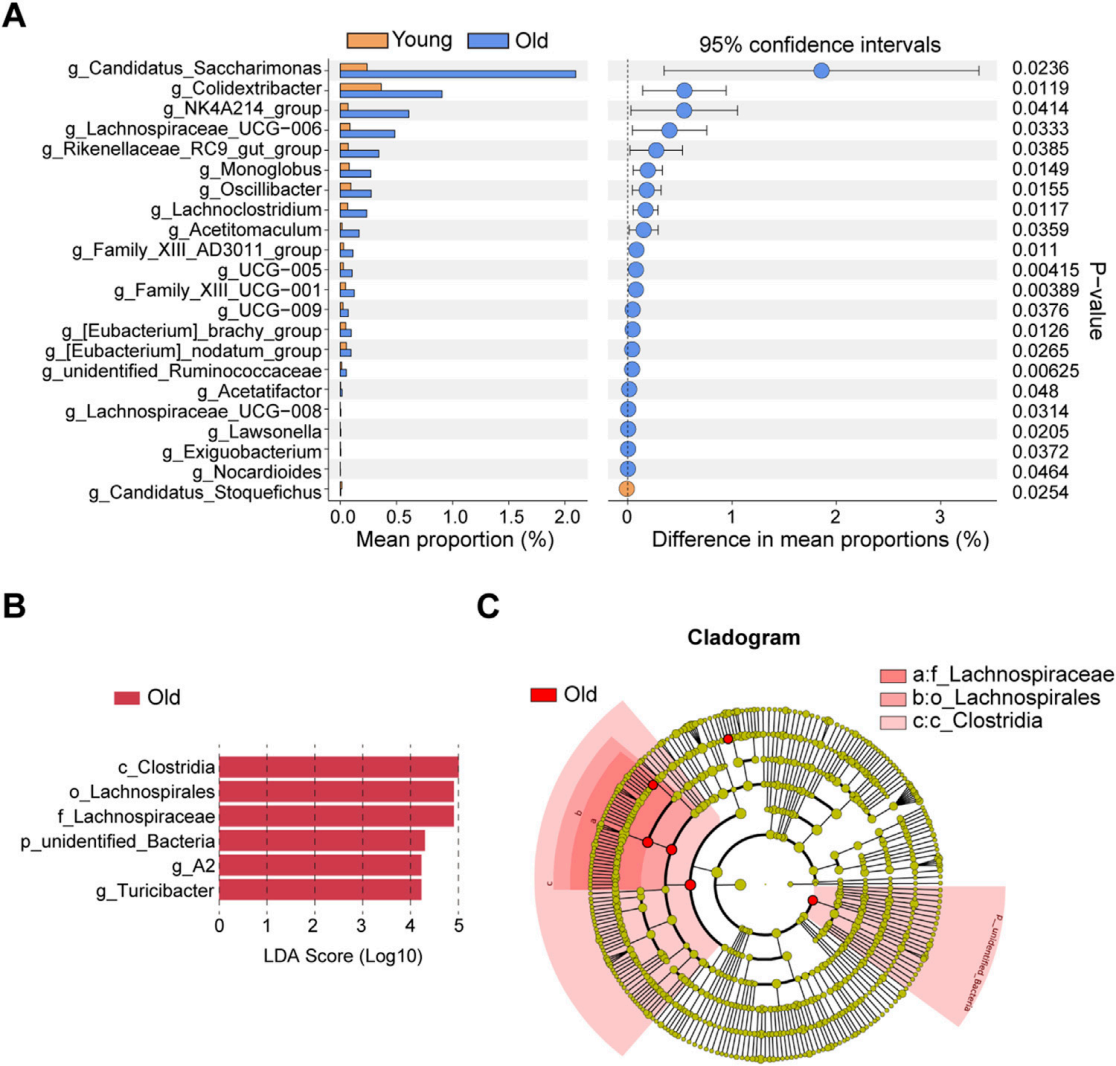
Identifications of differential microbial taxa between young and old group. **(A)** Differential microbial taxa at the genus level based on Welch’s *t*-test. **(B)** Differential bacterial taxa associated with aging were recognized by LEfSe algorithm. **(C)** Phylogenetic tree of differential microbial taxa recognized by LEfSe.

The differential microbiota taxa at the genus level between young and old mice were evaluated based on intergroup differential analysis using Welch’s *t*-test (Figure 5A). We identified 21 differential genera that were remarkably more abundant in the old group, whereas *Candidatus_Stoquefichus* was the sole differential genus significantly enriched in young mice. Recent studies suggest that *Candidatus Stoquefichus* is more prevalent in young mice and may play a role in reversing age-related changes (Yu et al., 2024). Among differentially abundant genera in old mice, we identified a variety of potentially harmful and opportunistic pathogenic genera, including *Oscillibacter* (Chen et al., 2019) and *Monoglobus* (Chen et al., 2022) associated with increased intestinal permeability abnormalities, the pro-inflammatory *Family_XIII_AD3011_group* (Zhang et al., 2021) and *Lachnoclostridium* (Cai et al., 2022), and pathogenic bacteria closely related to ischemic stroke and chronic kidney disease, viz., *Eubacterium_nodatum_group* (Chen et al., 2022). Two microbiota taxa at the genus level were identified by LEfSe, including *A2* and *Turicibacter* (Figure 5B). Figure 5C presents a cladogram depicting the phylogenetic distribution of these microbiota taxa. The differential genus *Turicibacter*, in particular, has been shown to exhibit pro-inflammatory characteristics (Ma et al., 2018), and its enrichment in the guts of elderly individuals has been reported (Liu et al., 2020).

To validate whether the aging-related variability in the abundance of differential genera is analogous to that in humans, we analyzed 16S rRNA data from healthy individuals of different age groups available in GMrepo database, with the PRJEB11419 dataset used for validation. After sample filtering, as mentioned in the methodology section, we acquired 16S rRNA sequencing data of 1,357 healthy individuals, with samples collected from 20 countries, primarily including the United States, the United Kingdom, Australia, and Canada. The selected samples were divided into four different age groups, followed by the analysis of associations of relative abundance of harmful and opportunistic pathogenic genera with aging. We limited our analysis to *Oscillibacter* and *Lachnoclostridium* because some microbiota taxa were not detected. Samples with missing data for the target genera were excluded, and the relative abundance of genera was statistically compared across different age groups. The trend of *Oscillibacter* with age is depicted in Supplementary Figure S2A, revealing higher relative abundance in the 56–83 age group compared to individuals in the 1–18 and 19–35 age groups. Similarly, *Lachnoclostridium* exhibited a close correlation with age, with higher abundance in the 19–35 age group compared to the 1–18 age group, and a trend of increase in the 36–55 age group compared with the 1–18 age group, while the difference was not statistically significant (*P* > 0.05) (Supplementary Figure S2B). Furthermore, to elucidate the correlation between the aforementioned harmful genera and diseases, we performed LEfSe analysis based on multiple disease datasets from the GMrepo database. As outlined in Supplementary Figure S2C, *Oscillibacter* was overrepresented in various autoimmune diseases such as ulcerative colitis, Crohn’s disease, rheumatoid arthritis, Behçet’s syndrome, ankylosing spondylitis, and colorectal cancer datasets. *Turicibacter* and *Lachnoclostridium* were also found to be significantly enriched in datasets of ulcerative colitis and irritable bowel syndrome, respectively.

Next, bacteria at the genus level associated with aging were identified using the LinDA R package, which revealed that 38 microbial genera were significantly overrepresented, while two were downregulated, in aged mice. The results are visualized in the volcano plot (Supplementary Figure S3A) and detailed information of all differential genera identified by LinDA is presented in Supplementary Table S1. Notably, Welch’s t-tests and LEfSe analysis. Specifically, the two genera identified by LEfSe (*A2* and *Turicibacter*) and 16 genera identified by Welch’s *t*-test were validated in LinDA’s results (Supplementary Figure S3B), further supporting the reliability of these findings.

### Investigation into the functional alteration of gut microbiota and their biological significance under aging conditions

To investigate the functional alterations in gut microbiota under aging conditions, phylogenetic investigation of communities by reconstruction of unobserved states (PICRUSt) were conducted to predict the functional profiles of microbiota, and subsequently analyzed their biological significance. The functional analysis revealed that aging significantly compromised functions related to genetic information processing and human diseases at the level 1 KEGG classification (Supplementary Figure S4A). Additionally, aging markedly increased cellular motility and environmental adaptation, while downregulating functions associated with replication and repair (Supplementary Figure S4B). These findings demonstrated that the weakening of DNA repair function during organismal aging may be correlated with changes in the relative abundance and functional alterations of microbiota. Furthermore, we detected a relative decrease in functions related to enzyme family and nucleotide metabolism (Supplementary Figure S4B), both of which are classified as level 1 KEGG metabolic hierarchy. It is well-documented that microbial metabolites play a crucial stage in influencing aging process and regulating various organs, potentially impacting host aging and longevity through diverse mechanisms.

At the level 3 KEGG classification (Supplementary Figure S4C), the old group exhibited downregulation of functions associated with various DNA repair pathways, including DNA repair and recombination proteins, nucleotide excision repair, and homologous recombination. Intriguingly, aging significantly altered the functional phenotypes related to bacterial motility proteins, bacterial chemotaxis, peptidoglycan biosynthesis, and flagellar assembly. We hypothesize that the gut microbiota of the old group may possess stronger individual and collective migration abilities, potentially contributing to changes in gut microbiota diversity and the prevalence of gut microbiota displacement in elderly individuals (Keegstra et al., 2022). Finally, variations were found in functions related to metabolic pathways such as Purine Metabolism, Pyrimidine Metabolism, and Glycosyltransferases.

### Aging-related decline in intestinal barrier function and its correlations with inflammaging

**FIGURE 6:**
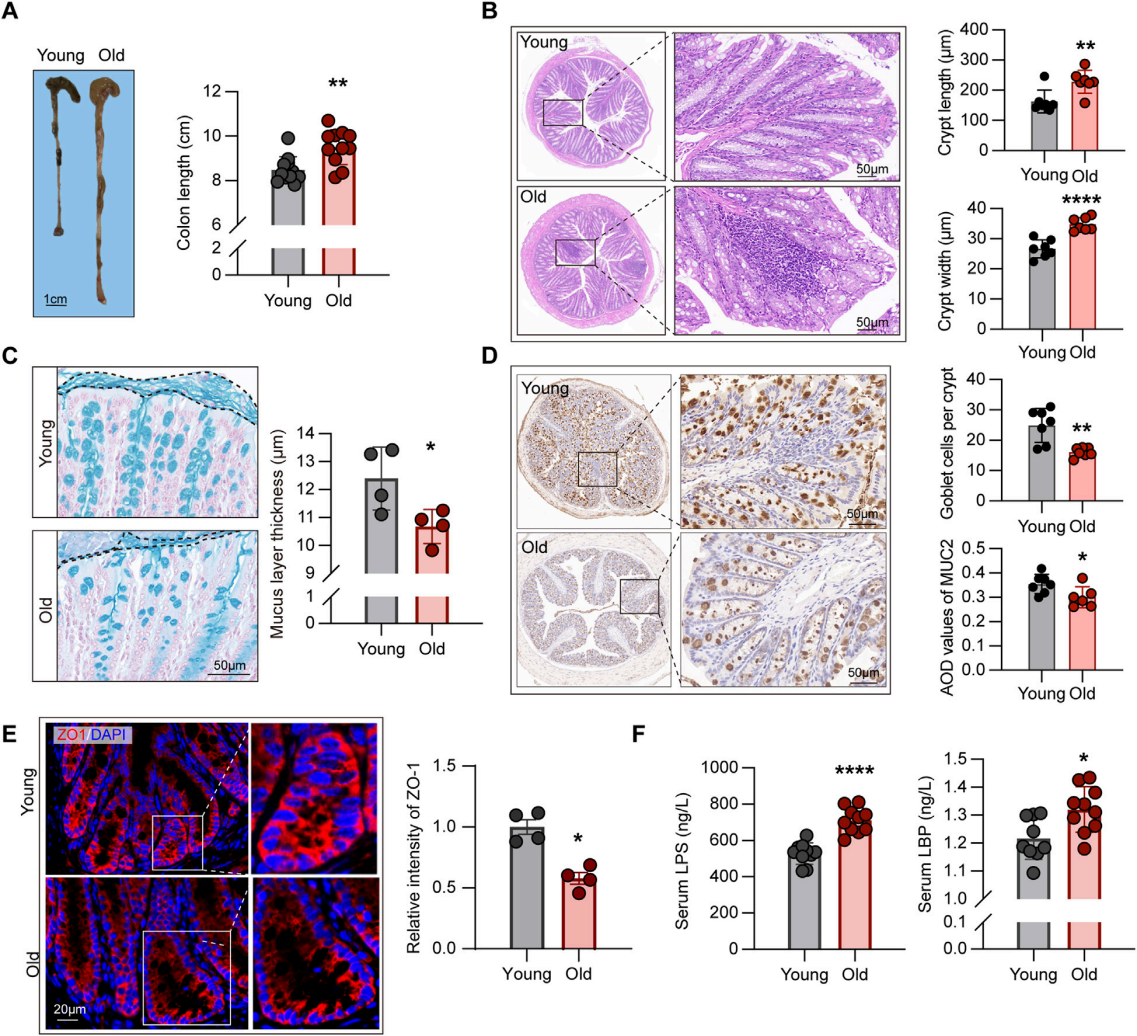
Aging-related alterations in intestinal structural and barrier function. **(A)** Comparison of length from colon to anus between young and old mice (n = 11). **(B)** HE staining showing crypt structures in the intestines of young and old mice, with at least 30 complete crypts analyzed per mouse (n = 7). **(C)** Comparison of mucus layer thickness in the intestines between young and old mice (n = 4). **(D)** IHC staining for MUC2 protein in intestinal sections from young and old mice, with average optical density (AOD) used to quantify staining intensity (n = 6–7). **(E)** Expression levels of ZO-1 protein in the intestines of young and old mice, measured using relative fluorescence intensity for group comparison (n = 4). **(F)** Comparison of plasma endotoxin levels in young and old mice (n = 9–10). *P < 0.05, **P < 0.01, and ****P < 0.0001.

Compelling evidence indicated that structural abnormalities and impaired barrier function in the intestines of aging individuals, contributing to the advent of “leaky gut” dysbiotic phenotype, yielding excessive immune activation and massive release of pro-inflammatory cytokines, namely inflammaging (Ragonnaud and Biragyn, 2021). Nevertheless, the correlation between microbiome alterations and related phenotypes remains elusive. To address this question, we first examined the morphological structures of mice of different ages. As such, we evaluated the length from colon to anus in each mouse (Figure 6A), indicating that this segment of the digestive tract was longer in old mice (*P* < 0.01).

Crypts are ecological regions containing various cell types (Hohman and Osborne, 2022), such as intestinal epithelial cells that maintain the integrity of epithelial barrier (Yoshimoto et al., 2021), goblet cells that produce mucin to form the mucus layer (Paone and Cani, 2020), and intestinal stem cells with differentiation capabilities (Barker et al., 2007). Subsequently, we examined the crypt structures of the intestines through HE staining. As depicted in Figure 6B, crypts of aging individuals were frequently accompanied by inflammatory cell infiltration, but those of young mice were overall intact. In addition, the crypts of the old mice were longer and wider than those of the young mice, according to the statistical analysis of both groups (Figure 6B), which contradicts the intestinal structural alterations found in mouse models of colitis and other diseases (Fan et al., 2021).

The intestinal barrier function is maintained by two layers of different structures, with the mucus layer formed by mucin-secreting goblet cells constituting the first essential line of defense against pathogenic microorganisms as well as various digestive enzymes (Ali et al., 2020). Alcian blue staining of the intestinal sections, as shown in Figure 6C, revealed the reductions in the thickness of the mucous layer in the old group. To corroborate the variances in the number of goblet cells between groups, we assessed the expression levels of MUC2 protein using IHC, with the results revealing the mitigated expression of such protein in the old mice (Figure 6D), consistent with the conclusions drawn from alcian blue staining. In addition, to the first barrier described above, the tight connections between intestinal epithelial cells constitute the second physical barrier, regulating the transport of ions, metabolites, and macromolecules outside the cells (Ali et al., 2020). Moreover, ZO-1 was assessed using IF, which demonstrated that the old group had lower ZO-1 expression levels than the young group (Figure 6E).

Subsequently, we assessed the levels of LPS in the serum of young and old mice. In Gram-negative bacteria, LPS forms a major component of the cell wall. Studies have demonstrated that increased intestinal permeability in aging individuals causes bacterial translocation, resulting in elevated levels of LPS in the circulation, which leads to excessive immune activation and the formation of an inflammaging state (Zhang et al., 2022). The ELISA findings revealed that the levels of circulating LPS in the old group were significantly higher than in the young mice (Figure 6F). Furthermore, we evaluated the levels of LBP in the circulation, which is a binding protein for LPS and is a crucial link in the downstream activation of inflammatory signaling pathways (Wang et al., 2021), and found a significant increase in circulating LBP levels in old mice (Figure 6F).

In this section, we systematically demonstrated aging-related changes in intestinal structure, along with 16S rRNA sequencing results, elucidating the mechanisms of inflammaging, and indicating that abnormalities in intestinal barrier function, resulting in microbiota translocation, endotoxins, and entry of various macromolecules into circulation, may be responsible for phenotypic alterations and increased susceptibility to diseases.

### Metabolome profiling and identifications of differential metabolites in young and old mice

**FIGURE 7:**
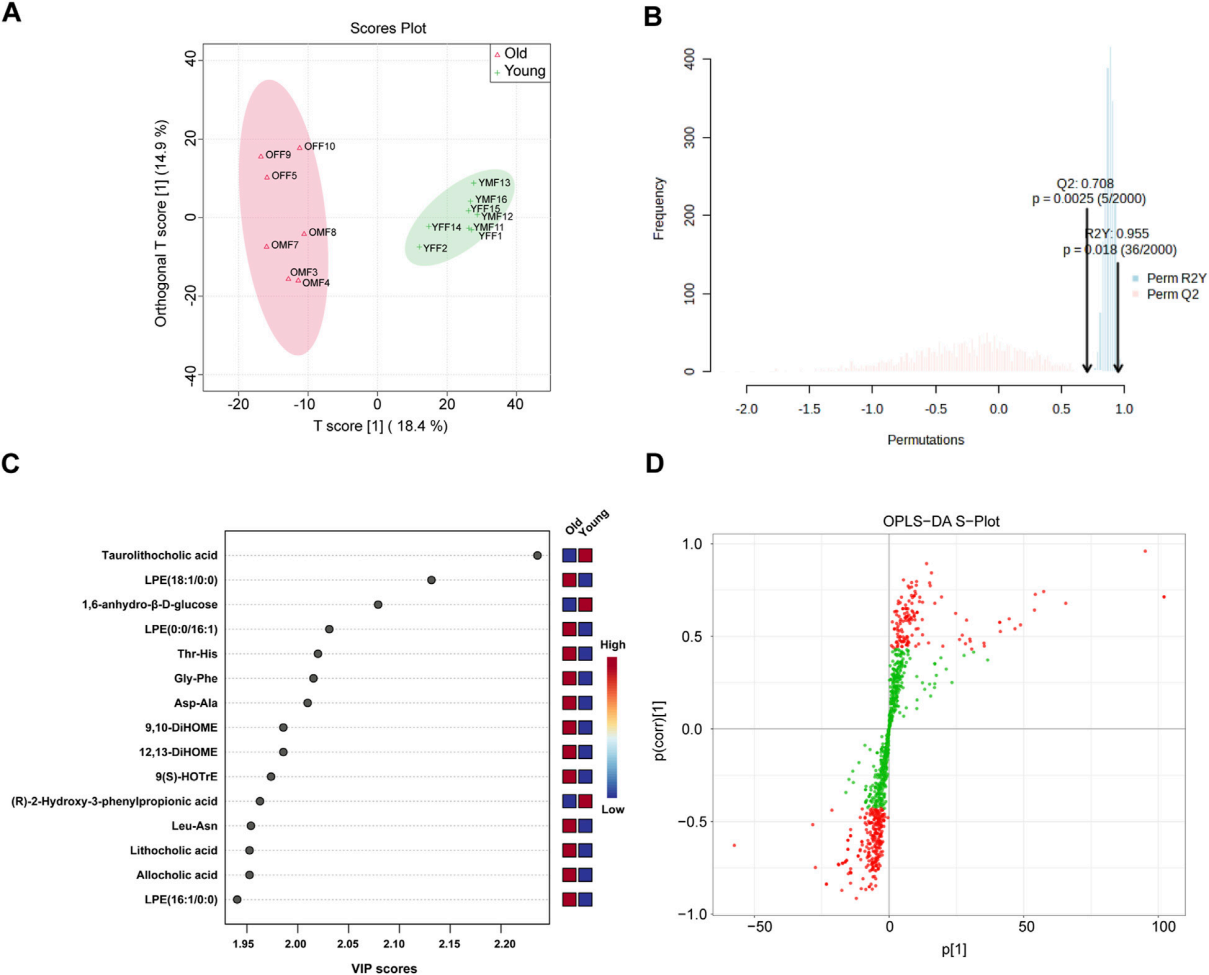
OPLS-DA of widely targeted metabolomic profiling of intestinal contents from young and old mice using UPLC-MS/MS. **(A)** The distribution of each sample in the young and old groups; **(B)** Permutation testing of OPLS-DA. **(C)** Top 15 metabolites ranked by VIP values in the OPLS-DA; **(D)** S-plot of OPLS-DA.

Our PICRUSt predictions of gut microbiota function revealed a significant age-related change in metabolic profile of gut microbiota. Therefore, widely targeted metabolomic profiling of intestinal contents was conducted from young and old mice using UPLC-MS/MS. Figure 7A displays significant differences in intestinal contents between groups as determined by OPLS-DA analysis. Subsequent model validation through permutation testing (n = 2000), Q^2^ = 0.708, R^2^Y = 0.955, *P* = 0.018, indicated good fitting accuracy of the OPLS-DA (Figure 7B). Figure 7C shows the top 15 metabolites ranked by their VIP scores in the OPLS-DA analysis. These metabolites exhibited higher VIP scores in the respective group, with taurolithocholic acid showing the highest VIP score in the young group. Figure 7D illustrates the S-plot of the OPLS-DA model, which provides a visual representation of the relationship between metabolites and the main components derived from the OPLS-DA analysis. Next, differential metabolites between different age groups were identified based on the absolute Log_2_FC ≥ 1 and VIP ≥1 as the threshold. Specifically, 63 metabolites were upregulated and 54 were significantly downregulated in the intestinal contents of old mice as visualized on volcano plots (Supplementary Figure S5). Next, the relative abundance of the 63 upregulated differential metabolites in the old group and their corresponding level 1 KEGG classification were displayed on a heatmap. The metabolites were primarily enriched in “lipid” and “amino acid” categories (Supplementary Figure S6A). Of note, we found that several secondary bile acids were significantly increased in old group, including “lithocholic acid”, “ursodeoxycholic acid”, “isolithocholic acid”, and “dehydrolithocholic acid”, indicating that aging may be accompanied by enhanced production of certain secondary bile acids to some extent.

Similarly, the 54 metabolites which were downregulated with age were visualized (Supplementary Figure S6B), most of which belonged to the “organic acids”, “amino acids”, “carbohydrates”, and “bile acids” of the level 1 KEGG classification. Interestingly, some of the metabolites downregulated in aged individuals are known to have anti-inflammatory or beneficial effects. For example, phytosphingosine possesses potent anti-inflammatory activity *in vitro* (Montenegro-Burke et al., 2021), taurolithocholic acid, which function as an anti-aging agent by improving mitochondrial function (Beach et al., 2015), indole-3-carbinol, which ameliorates gut dysbiosis phenotype in colitis mice models and increases the abundance of beneficial bacteria (Busbee et al., 2020), and indole-3-lactic acid, which inhibits the production of inflammatory cytokines and exerts dual anti-tumor effects (Sugimura et al., 2021).

### Enrichment analysis of differential metabolites and visualization of key pathways

To identify the signaling pathways influenced by these age-related metabolites, we performed KEGG enrichment analysis using the upregulated and downregulated metabolites. The results indicated that “starch and sucrose metabolism” may be the most significantly affected signaling pathways during the aging process (*P* < 0.05), with four differential metabolites associated with this pathway. Supplementary Figure S7A indicated that this pathway contained four differential metabolites, including Trehalose, Maltose, Trehalose, and UDP-Glucose, all of which were significantly downregulated in the old group (Supplementary Figure S7B). Notably, similar impairments in this pathway have been linked to metabolic dysfunctions in conditions like diabetes (Yuan et al., 2022), suggesting that disturbances in sugar metabolism may not only contribute to metabolic disorders but also be associated with aging. Furthermore, previous studies have demonstrated that trehalose exhibits neuroprotective effects in disease models, such as stroke (Forte et al., 2021). Collectively, our findings suggest that aging may significantly impair the gut microbiota’s ability to metabolize various sugars. This impairment could potentially contribute, to some degree, to an increased susceptibility to various aging-related diseases.

### Correlation analysis between aging-associated differential genera and metabolites

**FIGURE 8:**
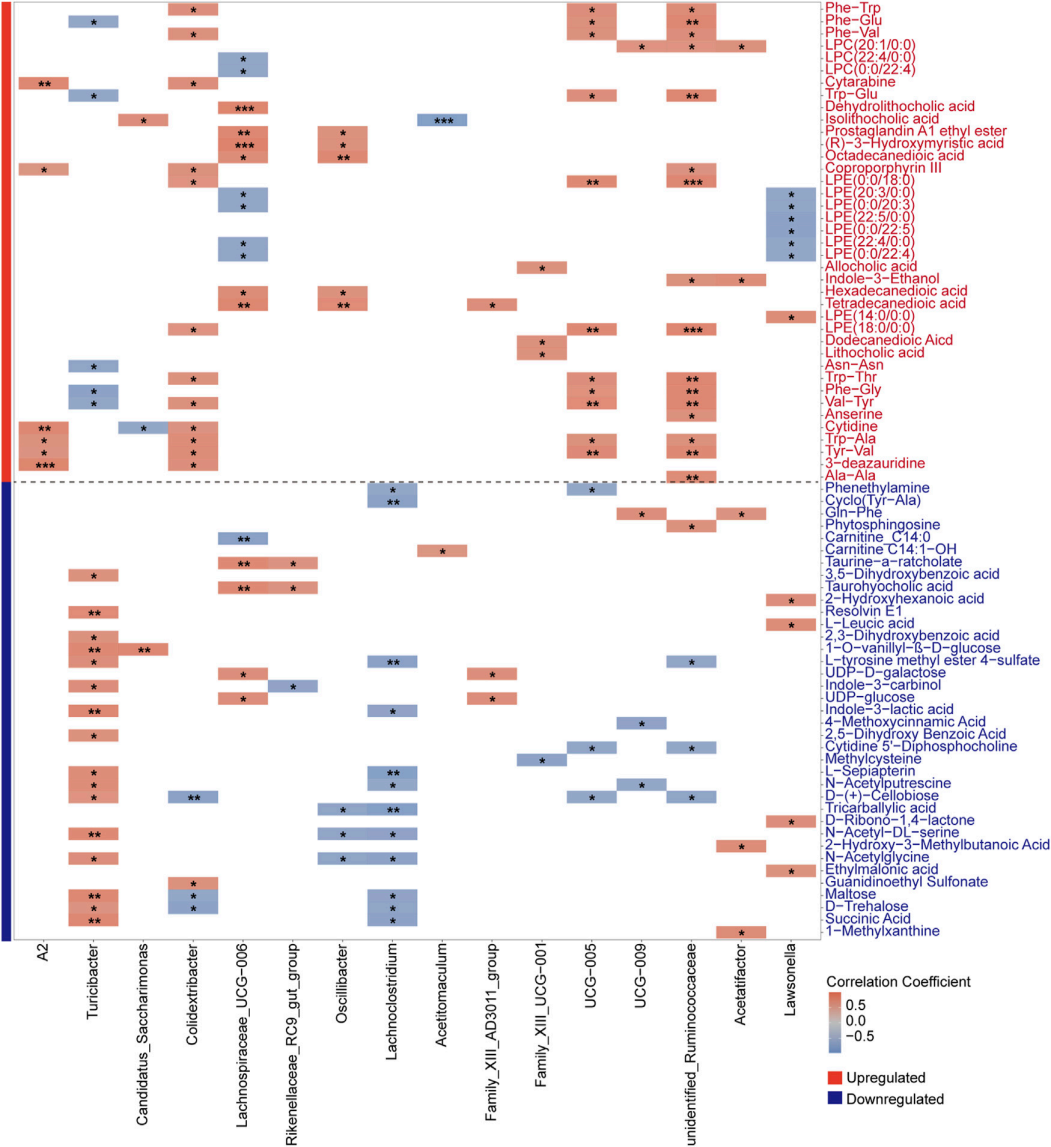
Spearman correlation heatmap showing associations between aging-associated bacterial genera and both upregulated (red-labeled) and downregulated (blue-labeled) metabolites in old mice. Differential genera, defined by the results from LEfSe and the intersection of LinDA and Welch’s *t*-test, were analyzed with Bonferroni correction to identify robust correlations. Significant correlations are indicated by *P < 0.05, **P < 0.01, and ***P < 0.001, with color intensity reflecting correlation strength.

After characterizing the metabolites in the intestinal contents of young and aged mice, we further explored their correlations with aging-associated differential genera by performing a Spearman correlation analysis. To address multiple testing, we applied the Bonferroni correction to control for potential false positives, thereby identifying robust associations between gut microbes and metabolites. This analysis specifically examined the correlations between differential bacterial genera and upregulated metabolites in old mice (Figure 8), with differential genera defined as those identified by LEfSe and the intersecting results from LinDA and Welch’s *t*-test. We observed a strong positive correlation between the aging-associated upregulated genus *Lachnospiraceae_UCG-006* and the secondary bile acid metabolite dehydrolithocholic acid, with a notably high correlation coefficient. Previous studies have highlighted the role of *Lachnospiraceae* in bile acid metabolism, suggesting that the increased abundance of this genus may be linked to elevated levels of secondary bile acids (Sorbara et al., 2020). Evidence suggests that excessive production of secondary bile acids may promote the accumulation of ROS and senescence-associated secretory phenotype (SASP), which has been implicated in the occurrence of various disease phenotypes (Yoshimoto et al., 2013).

Subsequently, we conducted Spearman correlation analysis between differential bacterial genera and downregulated metabolites in old mice (Figure 8). Mainly, we evaluated the correlation between downregulated metabolites in the Starch and Sucrose Metabolism pathway and the abundance of differential genera. Interestingly, we observed a significant association between the aging-upregulated, pro-inflammatory genera *Family_XIII_AD3011_group* and *Lachnoclostridium* with various downregulated metabolites from the Starch and Sucrose Metabolism pathway. We speculate that gut microbiota dysbiosis during aging may lead to altered abundances of bacteria carrying genes encoding carbohydrate-active enzymes.

## Discussions

According to projections by the WHO, the population aged above 65 will be the fastest-growing age group by 2050, and the proportion of this age group in the global population is expected to increase from 9% in 2020 to 16% in 2050. Population aging is a growing concern, and many countries around the world are now prioritizing proactive strategies to address it. Recent advancements in gut microbiota research are helping to shed light on the crucial role these microbes play throughout our lives. Notably, several factors affect gut microbiota, including diet, ethnicity, living environment, genetic factors, and even mode of delivery (Parizadeh and Arrieta, 2023), all of which can profoundly influence the compositions of various microbial communities. In addition, age is an important biological variable influencing gut microbiota (Salazar et al., 2017).

Here, our findings revealed a significant increase in alpha diversity within the gut microbiota of old mice, indicating an association between aging and microbial richness. The results of beta diversity analysis also demonstrated that aging is associated with changes in microbial communities. Additionally, we observed an enrichment of harmful and opportunistic pathogens in the aged group. Among them, the microbial genus *Family_XIII_AD3011_group* has been shown to possess strong pro-inflammatory abilities. Similar to our findings, Zhang *et al.* observed an increase in this bacterial genus in mice exposed to high benzene doses. Furthermore, they found a strong link between this genus and both the toxic reactions in the hematopoietic system and the levels of the pro-inflammatory cytokine interleukin-5 (Zhang et al., 2021). Similarly, Xiao et al. (2021) found that this genus was enriched in patients with cancer-related fatigue. Furthermore, *Lachnoclostridium* was found to be upregulated in diseases such as atherosclerosis (Cai et al., 2022), depression (Radjabzadeh et al., 2022), and colorectal cancer (Wang et al., 2019). Research by Cai et al. (2022) showed that *Lachnoclostridium* contains trimethylamine (TMA) lyase sequences, and produce TMA which increases the formation of atherosclerosis. TMA can be converted into trimethylamine N-oxide (TMAO) in the liver to stimulate immune-mediated inflammatory responses via metabolic reprogramming. Interestingly, function predictions of gut microbiota based on PICRUSt showed that the microbial genes related to “bacterial motility proteins”, “bacterial chemotaxis”, “peptidoglycan biosynthesis”, and “flagellar assembly” were enriched in old mice. Of particular interest, “bacterial chemotaxis” is a foraging strategy that allows bacteria to locate nutrient or energy sources. Current research suggests that this ability helps bacterial populations expand within their existing environment and even colonize new ones (Keegstra et al., 2022). Similarly, bacterial flagella facilitate rapid cell movement via rotation. Thus, we speculate that gut microbiota in aging individuals may exhibit enhanced migration capability, undergoing chemotaxis movement induced by metabolites circulating in the bloodstream (Dufrêne and Persat, 2020).

Furthermore, we observed structural alterations in the intestines of aging individuals. Analysis of intestinal tissues from the old group showed a decrease in goblet cell numbers, a thinner mucus layer, and disrupted tight junctions between epithelial cells. LPS is a molecule that is embedded in the cell wall of Gram-negative bacteria (Di Lorenzo et al., 2022). It can trigger strong inflammatory responses (Ghosh et al., 2015). Initially, LPS binds physically to LBP, which then transports LPS to toll-like receptor 4 (TLR-4) on the surface of immune cells via the membrane receptor CD14. This leads to activation of the immune system by inducing signaling factors such as myeloid differentiation factor 88 (MyD88) and interleukin receptor-associated kinase, promoting inflammatory response (Di Lorenzo et al., 2022). Collectively, LPS and LBP which are enhanced following microbiota displacement by impaired barrier function, indicate a connection between aging gut phenotypes and inflammaging.

Advances in gut microbiota profiling have yielded two key insights: the unique composition of microbial communities at various taxonomic levels, and the role of microbial metabolites in both healthy and disease processes within host. Within mucosal layer, microbial communities can affect the host by actively secreting metabolites or by-products derived from bacterial death, which contributes to the maintenance of local intestinal homeostasis (Zhou et al., 2021). Moreover, metabolites can passively cross the mucus layer of the intestinal epithelium and the barrier formed by tight junction proteins between intestinal epithelial cells, to regulate multiple organs (Li et al., 2018). From a physiological perspective, the proximity of the small intestine to the stomach creates local acidic and oxygen-rich environment, where Paneth cells secrete antimicrobial peptides, which limit bacterial growth, especially anaerobic bacteria. In contrast, in the colonic region, changes in the local environment increases the abundance of gut microbiota, thereby altering the metabolism of carbohydrates, dietary fibers, and resistant starches in the host (Donaldson et al., 2016). Gut microbiotas produce numerous metabolites by breaking down nutrients, including short-chain fatty acids (SCFAs), bile acids, and polyamines These metabolites are indispensable in maintaining host metabolic and endocrine homeostasis. Aging can alter the metabolites, which may serve not only as potential markers of the degree of aging but also as factors that can reverse aging phenotypes or treating age-related diseases. However, the metabolic alterations associated with the aging process are not well completely understood. Herein, we used widely targeted metabolomic profiling of gut microbiota from mice of various ages using UPLC-MS/MS. The impact of aging on the microbial metabolome was also investigated. Based on PCA and OPLS-DA, we observed significant differences in metabolic features between the young and old mice. In total, 63 metabolites were upregulated and 54 metabolites were downregulated in the old mice, belonging to ten different KEGG classifications.

Interestingly, several secondary bile acids, including lithocholic acid, ursodeoxycholic acid, isolithocholic and dehydrolithocholic acid, were increased in old group. Conversely, conjugated bile acids like taurolithocholic acid and taurohyocholic acid were downregulated in this group. Secondary bile acids are produced from primary bile acids excreted into the intestine under the action of gut microbiota through dehydrogenation and decarboxylation (Ridlon et al., 2006). Conjugated bile acids, formed by glycine or taurine attaching to free bile acids, reflect both the host’s physiological state and the composition of gut microbiota (Guzior and Quinn, 2021). Wei D. et al. (2022) demonstrated that the abundance of *Escherichia coli* increased significantly in old mice, which enhanced the degradation of tauroursodeoxycholic acid to taurochenodeoxycholic acid, the latter of which increase the production of TMAO in the liver, yielding hepatic fat accumulation. Various secondary bile acids associated with gut microbiota have been implicated in the occurrence of various diseases such as colorectal cancer (Cao et al., 2017), diabetes (Lamichhane et al., 2022), and renal failure (Wang et al., 2020). Undoubtedly, these results indicated that aberrant bile acid metabolism may reflect changes in aging and suggesting that the gut microbiota-bile acid regulation axis can be used to treat various age-related diseases.

Among the differential metabolites, we also observed disparities in the abundance of various tryptophan metabolites, including indole-3-lactic acid, indole-3-carbinol, and indole-3-ethanol. Tryptophan, an essential amino acid, can only be obtained through dietary intake. Once absorbed in the small intestine, specific bacterial strains expressing tryptophanase enzymes can break down tryptophan into indole derivatives within the gut. These metabolites play a role in the immune system by activating the aryl hydrocarbon receptor (AHR), promoting the host’s anti-inflammatory response, which modulates homeostasis of the host and gut microbiota (Roager and Licht, 2018). Studies have indicated that the expression level of proteins involved in metabolism decrease with age. Compared to infancy, a 50% decrease in relevant protein levels was observed between ages 11–31, and a decrease of over 90% was observed between ages 34–54 (Ruiz-Ruiz et al., 2020). This study revealed that old mice had significantly lower levels of indole-3-lactic acid and indole-3-carbinol compared to young mice. Interestingly, research suggests that specific bacteria found in breast milk and the infant gut can produce indole-3-lactic acid *in vitro* and may also exert anti-inflammatory effects on intestinal epithelial cells (Meng et al., 2020). Similarly, indole-3-carbinol was reported to have beneficial effects. For instance, indole-3-carbinol treatment improved local inflammatory responses in colitis mice, thereby increasing the abundance of Gram-positive bacteria producing SCFAs in the intestine (Busbee et al., 2020), and in a model of staphylococcal enterotoxin-induced liver injury (Busbee et al., 2015). Therefore, we postulated that alterations in tryptophan metabolism may occur during aging and promote the generation of inflammaging in a gut microbiota-dependent manner.

We systematically investigated global profile of gut microbiota during aging. Although our study has revealed important clues, there are some limitations that should be acknowledged. First, we did not establish a causal link between age-related alterations in gut microbiota or metabolites and inflammaging using functional experiments. Furthermore, aging, as a specific intermediate phenotype, may have specific effects on microbiota in certain diseases (such as cancer). However, we did not investigate the association between aging and diseases in this paper. Finally, there are differences between humans and mice, implying that mice-based studies may not accurately reflect changes in humans.

## Conclusion

Overall, this study aimed to provide insights into the complex interactions involved in organismal inflammaging through microbial multi-omics. These findings lay a solid foundation for future research aimed at identifying novel biomarkers for the clinical diagnosis of aging-related diseases or potential therapeutic targets.

## Data Availability

The original contributions presented in the study are included in the article/supplementary material, further inquiries can be directed to the corresponding authors.
