# Supplementary figures and images for: Multi-omics insights implicate the remodeling of the intestinal structure and microbiome in aging
Source: Front Genet. 2024 Nov 12;15:1450064. doi: 10.3389/fgene.2024.1450064 (PMC11588687; doi:10.3389/fgene.2024.1450064)

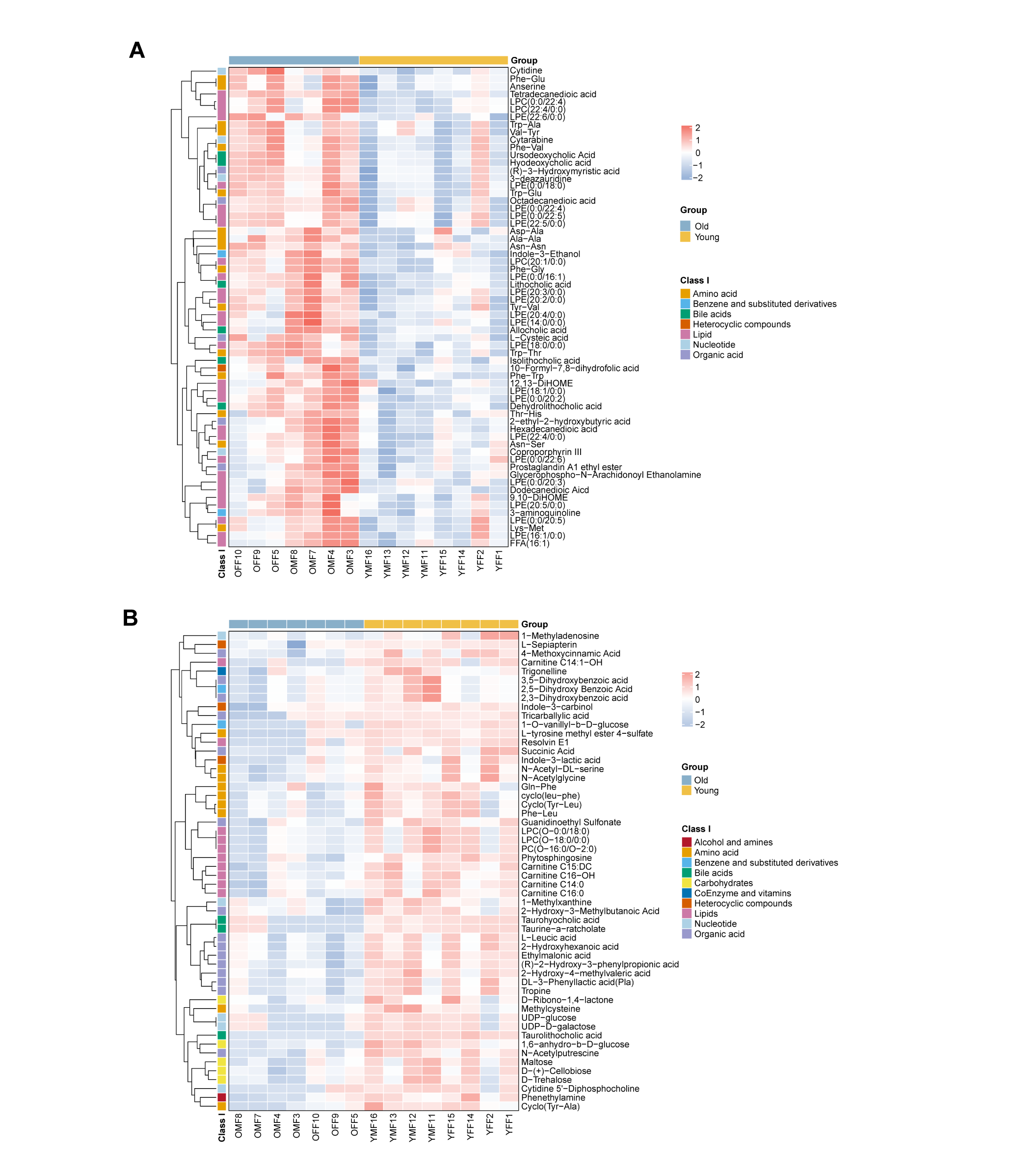

Supplement: Supplementary file 1 [file Image6.tif]

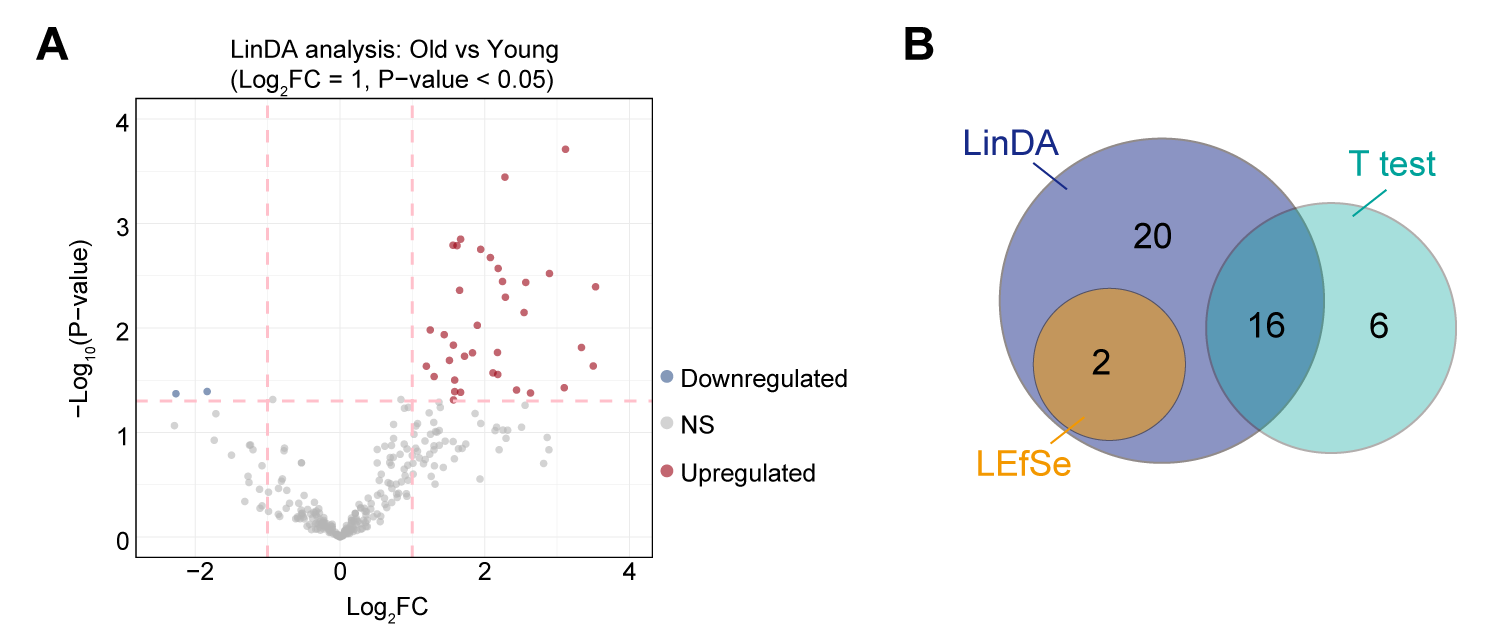

Supplement: Supplementary file 2 [file Image3.tif]

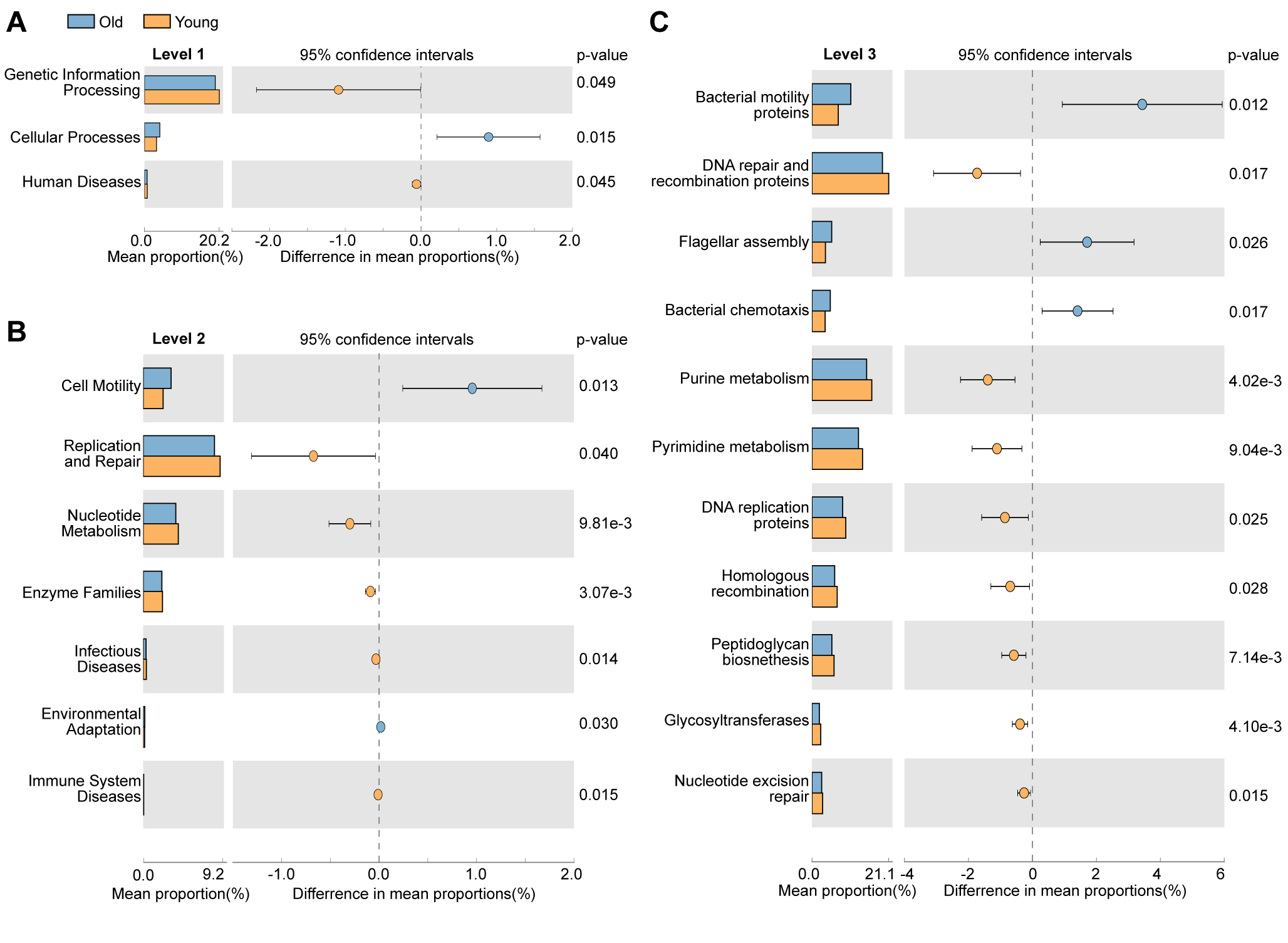

Supplement: Supplementary file 3 [file Image4.tif]

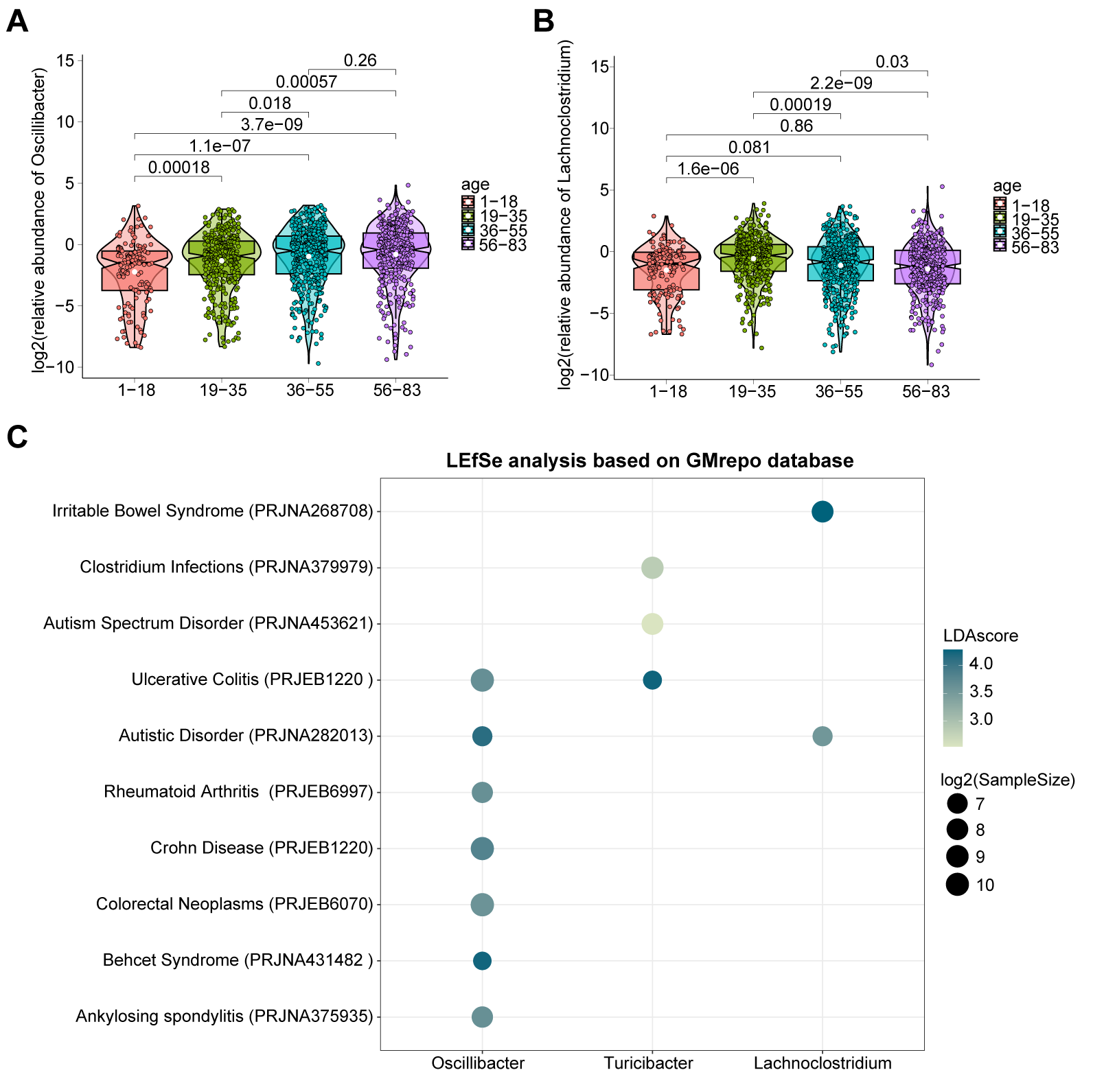

Supplement: Supplementary file 4 [file Image2.tif]

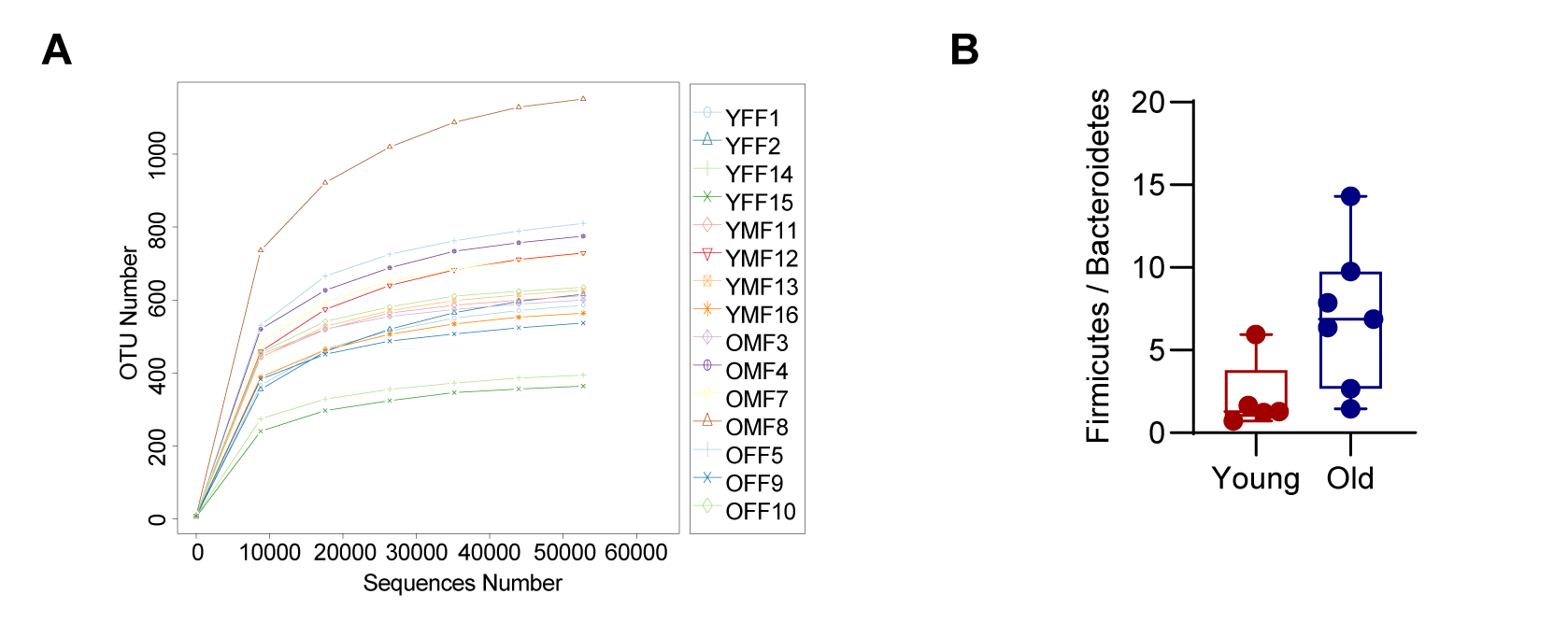

Supplement: Supplementary file 5 [file Image1.tif]

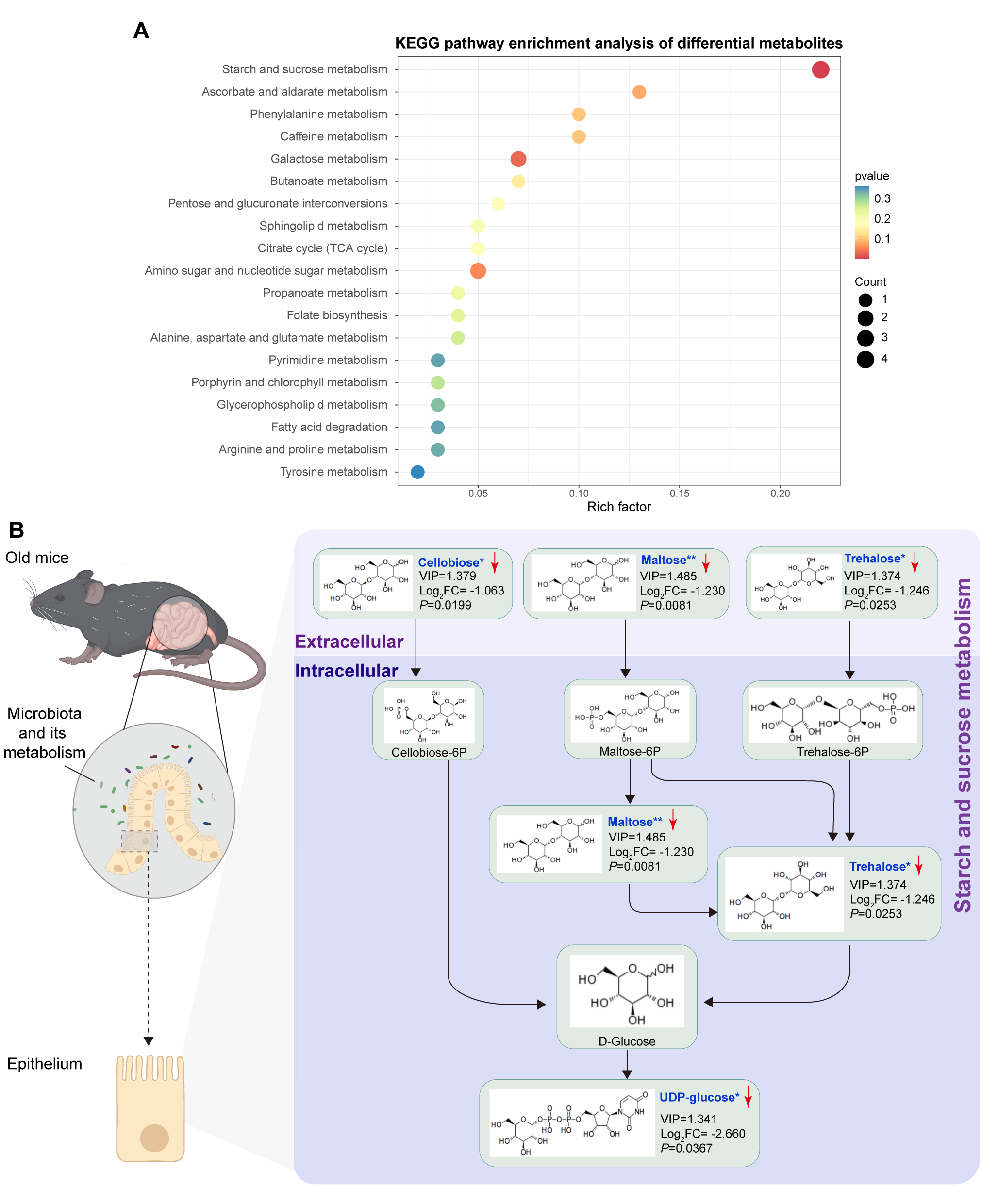

Supplement: Supplementary file 6 [file Image7.tif]

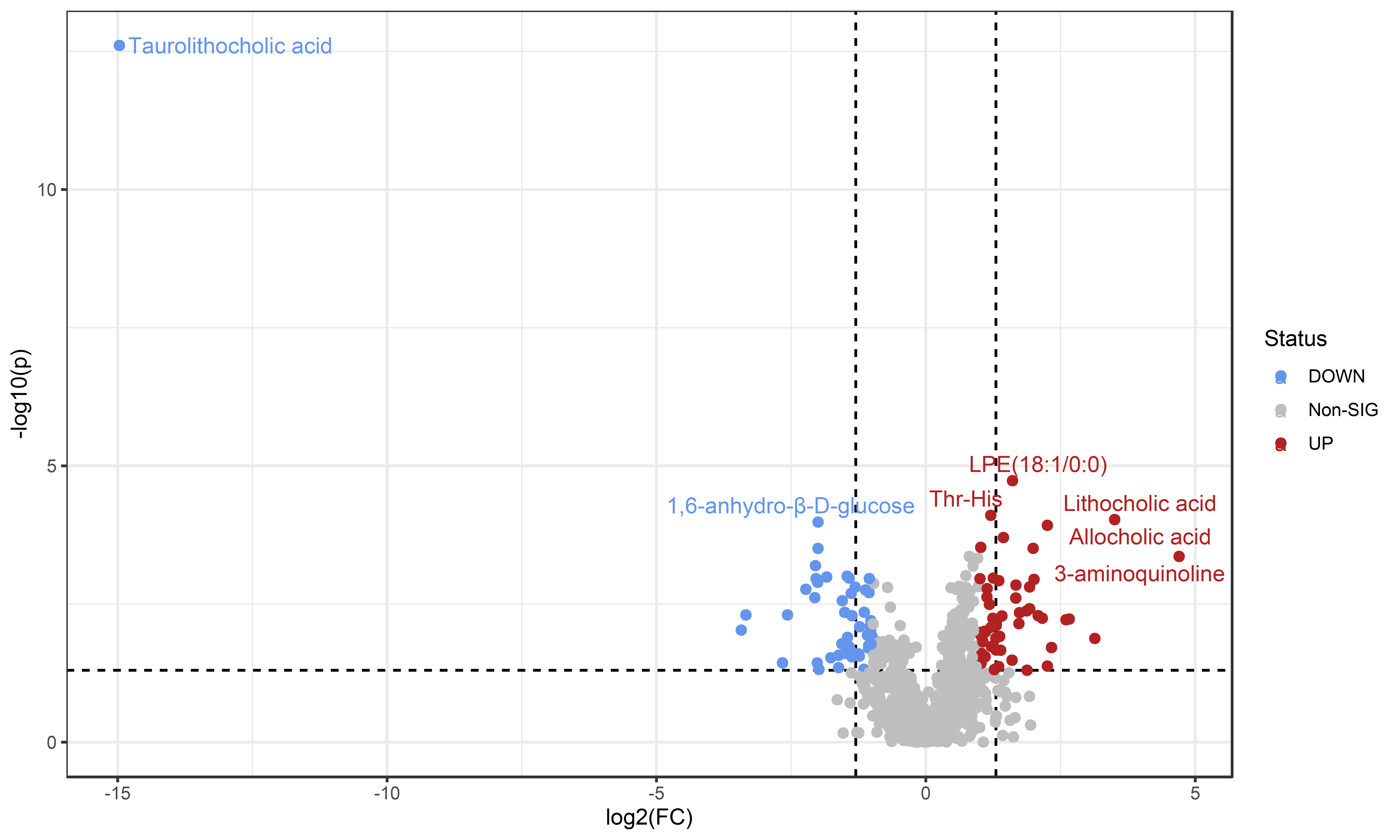

Supplement: Supplementary file 7 [file Image5.tif]
